# The age-related pattern of inner retinal thickening is affected by myopia development and progression

**DOI:** 10.1038/s41598-022-26598-w

**Published:** 2022-12-23

**Authors:** Reynolds Kwame Ablordeppey, Carol Lin, Alexandra Benavente-Perez

**Affiliations:** Department of Biological and Vision Sciences, College of Optometry, State University of New York, 33 West 42nd Street, New York, NY 10036 USA

**Keywords:** Experimental models of disease, Visual system

## Abstract

The longitudinal effect of myopic eye growth on each individual retinal layer has not been described to date on an established non-human primate (NHP) model of myopia. We evaluated the changes experienced by the overall and individual central and mid-peripheral retinal thickness profiles in marmosets (*Callithrix jacchus*) induced with myopia continuously for 5.5 months compared to controls using spectral-domain optical coherence tomography. Cycloplegic refractive state (Rx), vitreous chamber depth (VCD) and retinal thickness were measured at baseline and after 3 and 5.5 months on thirteen marmosets: eight animals with lens-induced myopia and five untreated controls. The overall and individual retinal layer thickness in the central and mid-peripheral retina were obtained and compared between groups. Regression models were used to explore the extent to which VCD or Rx changes could predict the thickness changes observed. While the retinas of control marmosets thickened significantly over 5.5 months, marmosets with lens-induced myopia experienced less retinal thickening and thinning at times, mostly in the inner neuroretinal layers and the ganglion cell-inner plexiform layer. The regression models suggest that 90% of the growth and refractive changes observed could be predicted by the thickness changes in the near to mid peripheral retina. This study confirms the longitudinal effect that myopia has on the inner retina of a NHP model during the early stages of myopia development. The observed myopia-driven differences in inner retina thickness templates might represent early biomarkers of myopia progression and associated complications.

## Introduction

Myopia has a unique signature on the retina that differs from normal physiological growth and can lead to significant visual consequences. The ease of its optical correction, however, can lead to an underestimation of its retinal implications. The increase in myopia prevalence and progressively younger age of myopia onset is predicted to result in a significant global increase in the number of patients suffering from high myopia and visual impairment^1,2^. Myopia, regardless of degree, is associated with ocular complications such as an increased risk of primary open angle glaucoma^3^, chorioretinal degeneration^4^, cataract^5^, staphyloma^6^ and retinal detachment^7^. Despite the major public health concerns associated with its rise in prevalence, severity, associated complications and socio-economic burden^8^, the mechanisms underlying the blinding ocular complications associated with myopia remain largely unknown.

During myopia development and progression, the eye accelerates its growth resulting in mechanical stretch and potential thinning of the ocular structures. Studies exploring the effect of ocular growth on the retinal architecture have described a lower cone density^9,10^ and thickness changes in the retinal nerve fiber and ganglion cell layers in cross-sectional clinical studies and longitudinal studies performed in humans^11–16^, chicks^17,18^ and tree shrews^19^. Most evidence points towards a regional retinal thinning that is proportional to the amount of axial length elongation. The thinning percentage and location, however, vary across studies and myopia degree. High myopic human eyes have significant foveal and temporal retinal nerve fiber layer thickening while also exhibiting thinning in other retinal quadrants compared to moderate and low myopes^16,20,21^. To fully understand the extent to which myopia development and progression affect the central and mid-peripheral retina thickness, we aimed to define the longitudinal effect of myopic eye growth on each individual retinal layer in non-human primate (NHP) eyes, which remains unknown to date.

Experimental animal models of myopia have advanced myopia research, improved our understanding of myopia mechanisms and helped develop clinical interventions for myopia control^22^. The common marmoset (*Callithrix jacchus*) is a new-world primate with visual optics matching those of humans: foveated retina, high visual acuity, dense cone photoreceptor distribution, large accommodative amplitudes, shallow depth of focus, and their ocular growth has been studied extensively^23,24^. Key elements involved in myopia development in humans are found in marmosets: circadian rhythm^25^, choroidal thickness changes^26^, accommodative behavior to changing defocus^27^, and similar peripheral refractive profiles^28,29^.

*In-vivo* visualization of each individual retinal layer using optical coherence tomography (OCT) provides an important source of information to understand the effect that myopia development and increased eye growth have on retinal morphology. Previous work in human and animal models describing this association have been cross-sectional or over a relatively short time course which does not allow for full comprehension of how the retina changes over time during myopia development and progression. We addressed this gap by studying the retinal thickness changes experienced by myopic eyes using our well-established marmoset model of myopia^28,29^.

In this study, we performed a detailed characterization of the effect that normal eye growth has on the retinal thickness of marmoset eyes longitudinally over 5.5 months compared to the effect that myopic eye growth has on the overall and individual layer thickness profiles, using spectral-domain optical coherence tomography (SD-OCT). By characterizing the changes that occur during the development of high myopia prior to the development of myopic complications, we aim to identify early markers of myopia progression that will open the field to preventive and intervention strategies for high myopia and associated diseases.

## Methods

### Experimental design

Thirteen juvenile marmosets (age: 68.92 ± 2.10 days) were studied longitudinally for 22 weeks: eight were treated binocularly (2 males M; 6 females F) for 22 weeks (5.5 months) using negative single-vision soft contact lenses to induce moderate and high degrees of myopia; five were untreated controls (4 M; 1 F). Statistical power analysis of the principle methods used indicated that 12 marmosets provide 80% power. Both experimental and control groups experienced an average of 9 h light (≈700 lx)/15 h dark cycle following our established protocol^29–31^. All animal care, treatment, and experimental procedures were approved by the SUNY College of Optometry Institutional Animal Care and Use Committee and complied with ARRIVE guidelines and the ARVO Statement for the Use of Animals in Ophthalmic and Vision Research. Based on the onset of marmoset sexual maturity (approximately 15 months old^32^), 1 month in the lifespan of a marmoset would be equivalent to 1 year in humans.

Contact lens treatment started at 70 ± 7 days of age using -5D contacts in both eyes. The treatment power was increased to -10D once marmosets compensated for -5D. Corneal curvature was measured with a custom-made infrared video keratometer, and lenses were fitted 0.10 mm flatter than the flattest k measure, following our established lens-rearing paradigm^28,29,31^. All soft contact lenses were made from methafilcon A (58% water content, oxygen permeability: 21, from Capricornia Contact Lens, Pty Ltd, Queensland, Australia). No ocular complications due to contact lens wear were observed in any marmoset in this or previous studies.

### Outcome measures

Refractive state (ARK-900 autorefractor, Nidek Co., Ltd, Gamagori, Japan), ocular biometry (A-scan ultrasound 25 MHz, Panametrics; NDT, Ltd, Waltham, MA, USA) and SD-OCT (Bioptigen, Inc., Durham, NC, USA) were assessed at baseline (start of treatment; T1), after 12 weeks (mid-treatment; T2) and 22 weeks of treatment (end of treatment; T3) to obtain measures of refractive error (Rx), vitreous chamber depth (VCD) and retinal thickness, respectively. Cycloplegic refractive error was obtained from awake animals (1% cyclopentolate), after which animals were anesthetized with alphaxalone (15 mg/kg, I.M.) to perform ocular biometry and SD-OCT. Each OCT scan was a rectangular volume retinal scan (12 × 5.40 × 12mm^3^, 700 A-scans/B-scan × 70 B-Scans, 2048 voxels per A-scan) with 5 frames at each B-scan location. The 5 frames/B-scan were averaged to reduce speckle noise and improve the signal-to-noise ratio. To further enhance image quality, marmosets wore custom-made rigid contact lenses (3.75 mm base curve, 5 mm diameter, 0.00D refraction, Conforma Laboratories, Inc., Norfolk, VA, USA) and artificial tears was applied to prevent tear film evaporation during the OCT imaging. All measurements were performed between 9 to 11am to control for the diurnal variations in ocular parameters known to occur in marmosets^33^.

**Figure 1:**
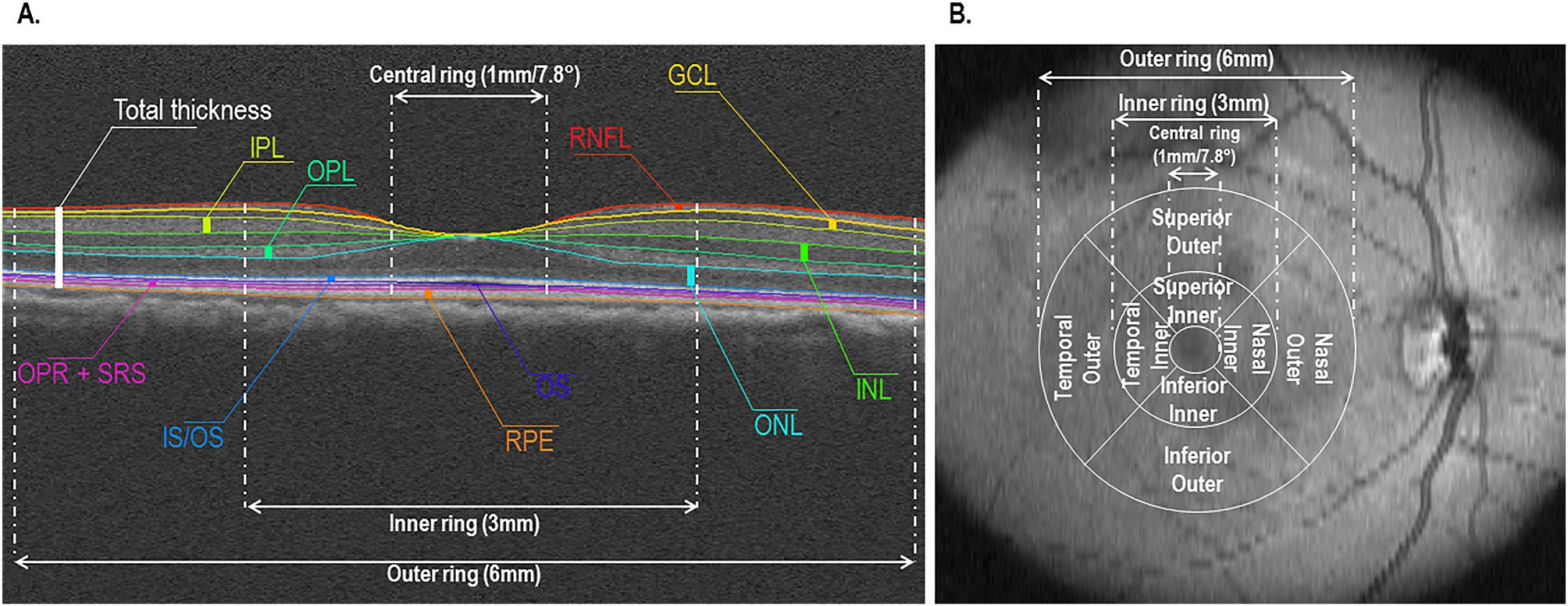
(**A**) Automatic macular thickness segmentation of a cross-sectional OCT scan of a marmoset retina in XZ layout. (**B**) The layout of the ETDRS grid on an *en-face* marmoset image. RNFL = retinal nerve fiber layer; GCL = ganglion cell layer; IPL = inner plexiform layer; INL = inner nuclear layer; OPL = outer plexiform layer; ONL = outer nuclear layer; IS_OS = inner segments/outer segments layer, OS = outer segment, OPR = outer segment photoreceptor + subretinal virtual space, and RPE = retinal pigment epithelium.

We used the OCT segmentation and quantification protocol previously developed and validated by our lab^34^ with the Iowa Reference Algorithms v3.8.0 (Retinal Image Analysis Lab, Iowa Institute for Biomedical Imaging, Iowa City, IA, USA) to segment the scans and obtain overall retinal thickness measurements, as well as the thickness of each individual retinal layer: retinal nerve fiber layer (RNFL), ganglion cell layer (GCL), inner plexiform layer (IPL), inner nuclear layer (INL), outer plexiform layer (OPL), outer nuclear layer (ONL), inner segments/outer segments layer (IS_OS), outer segment (OS), outer segment photoreceptor + subretinal virtual space (OPR), and retinal pigment epithelium layer (RPE) (Fig. 1A)^35^ The scans were visually inspected and segmentation errors were manually corrected.

The Early Treatment Diabetic Retinopathy Study (ETDRS) grid was used to obtain central thickness profiles in each of the nine regions defined by three concentric rings: central 1 mm diameter (fovea), middle 3 mm diameter (para and perifoveal retina) and outermost 6 mm diameter (near-mid peripheral retina) (Fig. 1B). The same foveal location was identified by choosing the scan with the deepest foveal pit and presence of foveal reflex. To minimize errors, only one researcher (RKA) selected the scans.

Thickness changes were calculated as the retinal thickness at mid-treatment or end of treatment minus that at baseline in each retinal layer. In addition, we also assessed whether the inner and outer retina changed differently as eyes grew normally or developed myopia. We defined the inner retina as the region from the internal limiting membrane to the external limiting membrane (RNFL to ONL), and the outer retina from the external limiting membrane to Bruch’s membrane (IS_OS to RPE). We also explored thickness changes in the Ganglion Cell Complex (GCC = RNFL + GCL + IPL) and Ganglion Cell-Inner Plexiform Layer (GCIPL = GCL + IPL), as these profiles are routinely used in clinical settings^36^.

To correct for myopic ocular magnification, the diameter of the ETDRS grid circle was adjusted to account for eye growth differences between control and treated eyes. For an emmetropic (0D) marmoset (average axial length: 9.62 mm), the standard central, middle and outermost ring radii were used: 0.5, 1.5 and 3 mm. In other controls and treated marmosets, the modified radii (R_N_) were calculated using a tangent function, where R is the radius of the ETDRS grid and AL is the axial length of the marmoset:

$$R_{N} = \frac{R \times AL}{{9.62}}$$

Prior to the longitudinal collection of data, Bland–Altman analyses evaluated the repeatability of the retinal acquisition and segmentation thickness calculations using two repeated scans from 15 marmosets (different from the 13 study marmosets).

### Statistical analyses

Data are described as mean ± standard deviation (SD). Data were collected from both eyes of each animal and only data from one random eye analyzed to avoid inter-eye correlations. Statistical analyses were performed using IBM SPSS Statistics for Windows (version 23.0; IBM Corp., Armonk, NY, USA). Data was assessed for normality using the Shapiro–Wilk test, and the appropriate statistical test was used. Repeated measures analysis of variance (ANOVA) was used to test the effect of emmetropization/time on retinal thickness in controls over time as well as the effect of treatment method (treated vs control) on refraction, vitreous chamber depth and retinal thickness changes over time. A statistically significant result was considered as *p*-value < 0.05. Stepwise multiple regression models were used to assess whether the changes in vitreous chamber depth and refractive error over the 22 weeks could be predicted by the retinal thickness changes observed. In these models, refractive error and vitreous chamber depth were the dependent variables, and the thickness measures in each ETDRS region and layer were the independent variables.

## Results

### Intrasession repeatability of OCT measurement and segmentation

**Figure 2:**
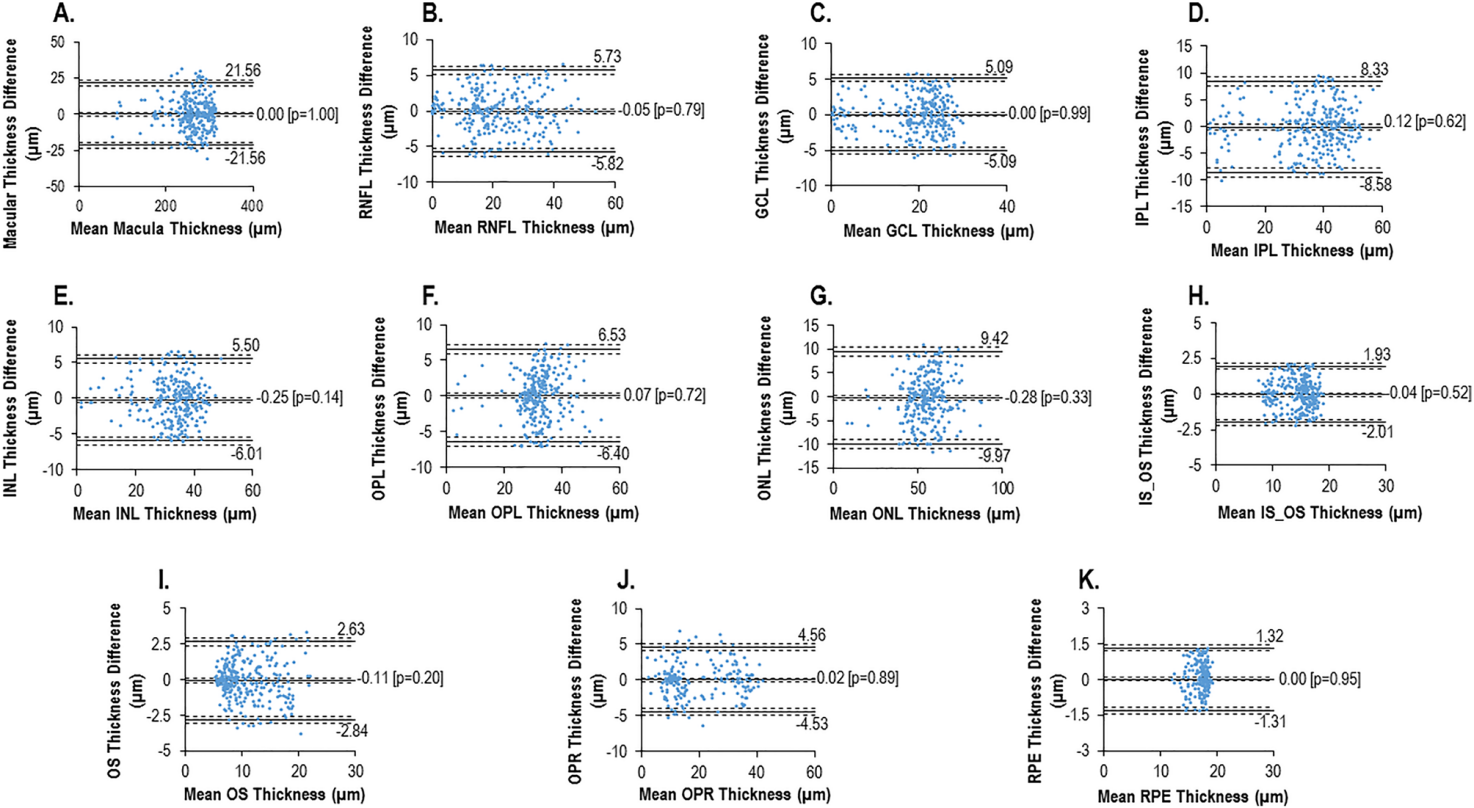
Bland–Altman plots examining the repeatability of the retinal thickness measures from two repeated scans within a session from each marmoset where the difference between the repeated scans is plotted against the mean of the two repeated scans. The solid lines in each plot indicates the mean difference and limits of agreement (LOA: ± 1.96 times the SD of the mean difference), while the dash lines represents the 95% upper and lower confidence interval of the solid lines (mean difference and LOA).(**A**) Overall retinal thickness, (**B**) RNFL, (**C**) GCL, (**D**) IPL, (**E**) INL, (**F**) OPL, (**G**) ONL, (**H**) IS/OS, (**I**) OS, (**J**) OPR + SRS, and (**K**) RPE.

The Bland–Altman analysis (Fig. 2) resulted in a mean thickness difference close to zero for the overall thickness and each individual retinal layer (range: − 0.28–0.07 µm). There were no significant differences between two repeated scans and their associated segmentations, neither for the overall thickness measures (*p* = 1.00) nor for each individual retinal layer (all layers; *p* > 0.05).

### Refraction, biometry and retinal thickness at baseline

At baseline before treatment started, treated and control animals did not differ in refractive state (Rx, treated: − 0.97 ± 0.91D, control: − 0.63 ± 0.60D, *p* = 0.48), eye size (VCD, treated: 5.78 ± 0.12 mm, control: 5.87 ± 0.12 mm, *p* = 0.19) or overall retinal thickness (treated: 256.27 ± 15.88 µm, control: 244.87 ± 16.22 µm, *p* = 0.24).

**Figure 3:**
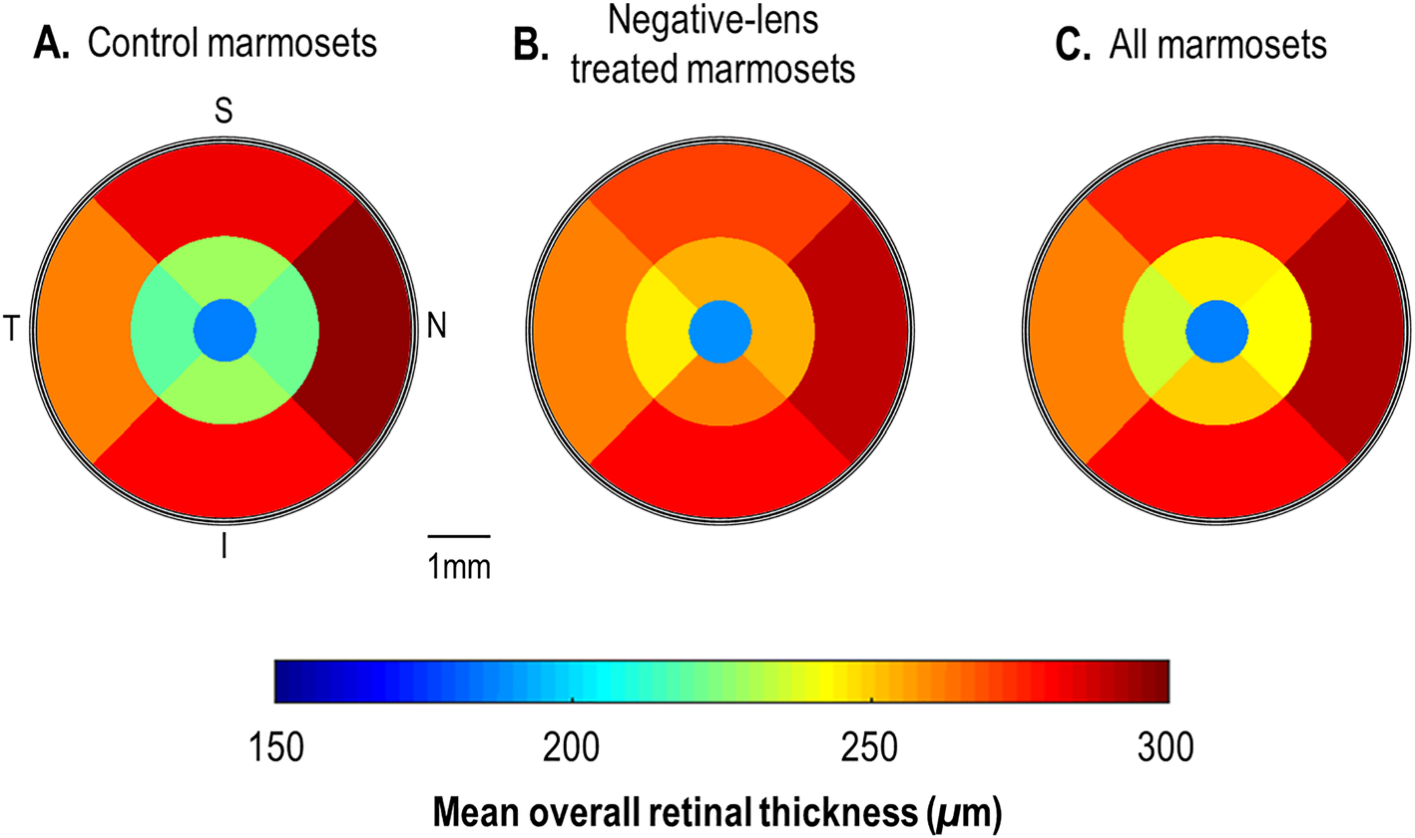
ETDRS color map summarizing the baseline mean overall retinal thickness measures for (**A**) control marmosets (*n* = 5), (**B**) negative-lens treated marmosets (*n* = 8) and (**C**) all marmosets in the two groups (*n* = 13) treatment groups. Control and negative-lens treated marmosets had similar overall retinal thickness profile at baseline. S = Superior; T = Temporal; N = Nasal; and I = Inferior.

**Figure 4:**
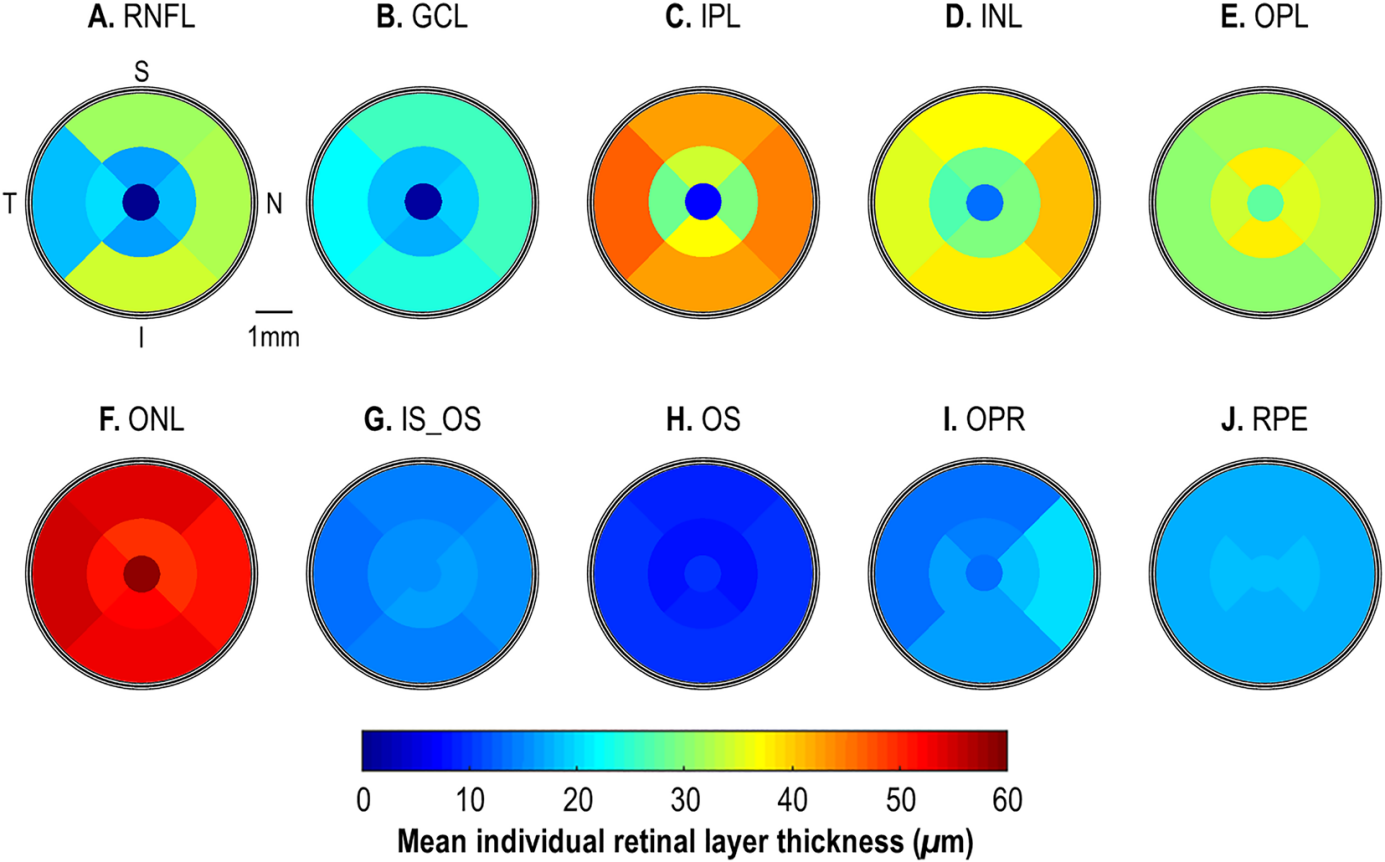
ETDRS color map summarizing the mean individual 10 retinal layer thicknesses for all common marmosets (n = 13) at baseline. S = Superior; T = Temporal; N = Nasal; and I = Inferior. RNFL = retinal nerve fiber layer; GCL = ganglion cell layer; IPL = inner plexiform layer; INL = inner nuclear layer; OPL = outer plexiform layer; ONL = outer nuclear layer; IS_OS = inner segments/outer segments layer, OS = outer segment, OPR = outer segment photoreceptor + subretinal virtual space, and RPE = retinal pigment epithelium. ETDRS color map summarizing the average baseline thickness for each individual retinal layer (**A**–**J**) for all marmosets (n = 13). S = Superior; T = Temporal; N = Nasal; and I = Inferior. RNFL = retinal nerve fiber layer; GCL = ganglion cell layer; IPL = inner plexiform layer; INL = inner nuclear layer; OPL = outer plexiform layer; ONL = outer nuclear layer; IS_OS = inner segments/outer segments layer, OS = outer segment, OPR = outer segment photoreceptor + subretinal virtual space, and RPE = retinal pigment epithelium.

There were no statistically differences in retinal thickness between the two experimental groups in any retinal region (*p* > 0.05; Fig. 3A,B). The overall average retina for all marmosets was thinnest in the center (187.02 ± 15.47 μm), followed by the temporal (248.26 ± 19.35 μm), superior (260.20 ± 16.18 μm) and inferior regions (264.37 ± 19.71 μm) and was thickest nasally (267.17 ± 18.83 μm) (Fig. 3C). In terms of the individual layers, the superior region was thicker than the inferior region in the GCL, OPL and RPE along the vertical meridian, whereas for the horizontal meridian, the nasal region was thinner than the temporal region in IPL and ONL. Figure 4 summarizes the topographical profile of each individual retinal layer thicknesses for all marmosets at baseline.

Similarly, there were no differences between treatment groups at baseline in terms of eccentricity (central ring, treated: 187.94 ± 19.60 μm, control: 185.55 ± 6.43 μm; *p* = 0.76; middle ring, treated: 254.07 ± 19.19 μm, control: 224.15 ± 24.37 μm, *p* = 0.05; outermost ring, treated: 275.57 ± 13.63 μm, control: 280.42 ± 13.71 μm, *p* = 0.55; Fig. 3A,B). For all marmosets, the retina was thickest at the most eccentric locations (overall retina; central ring: 187.02 ± 15.47 μm; middle ring: 242.56 ± 25.34 μm; outermost ring: 277.44 ± 13.30 μm; one-way ANOVA, *p* < 0.001; Fig. 3C). This pattern was observed in all inner retina layers except the OPL (middle ring > outermost ring > central ring) and the ONL (central ring > outermost ring > middle ring). In the outer retina, the retina was thickest in the central ring of the OS and RPE, but thinnest at the OPR. The IS_OS was thinnest and thickest in the outermost and middle ring respectively (Fig. 4).

### Effect of lens treatment on refraction and eye growth

**Figure 5:**
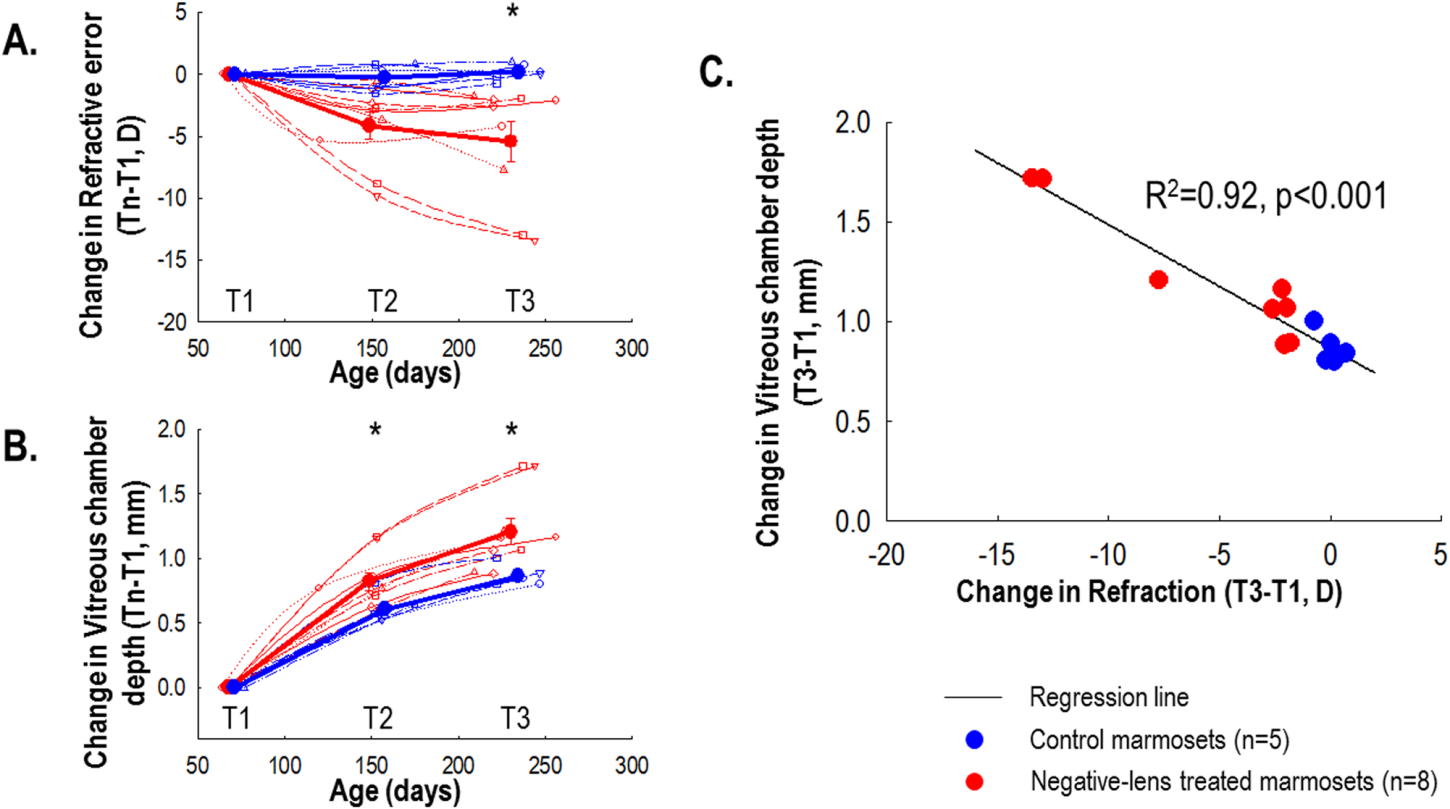
(**A**) Refractive error changes and (**B**) vitreous chamber depth changes, normalized to baseline, for control and negative-lens treated eyes in each group over the treatment duration. Unfilled thin symbols represent data for the individual marmoset whiles thick filled circles represent the mean for each group: control (blue) and negative-lens treated (red). Positive and negative values on the ordinate of Fig. 3A represent hyperopic and myopic refractive change relative to baseline, respectively. Statistically significant differences at each treatment time point are indicated by *p < 0.05. The data are shown as mean ± SE. (**C**) Change in vitreous chamber depth as a function of change in refraction after 22 weeks of treatment. Solid black line represents the regression line, while blue and red symbols are data for control and negative-lens treated marmosets, respectively.

Repeated measures ANOVA showed a significant time and treatment interaction effect on refractive error (*p* < 0.05; Fig. 5A) and vitreous chamber depth (*p* < 0.001 for time, *p* < 0.05 for time and treatment method interaction; Fig. 5B).

After 22 weeks of negative contact lens wear, treated marmosets developed axial myopia (treated Rx change: − 5.59 ± 5.08D, control: − 0.01 ± 0.54D; *p* < 0.05; treated VCD change: + 1.22 ± 0.33 mm, control: + 0.87 ± 0.08 mm; *p* < 0.05). The growth and refractive changes after 22 weeks were significantly associated (*R*^2^ = 0.92, *p* < 0.001; Fig. 5C).

### Effect of emmetropization on retinal thickness of control marmosets

**Figure 6:**
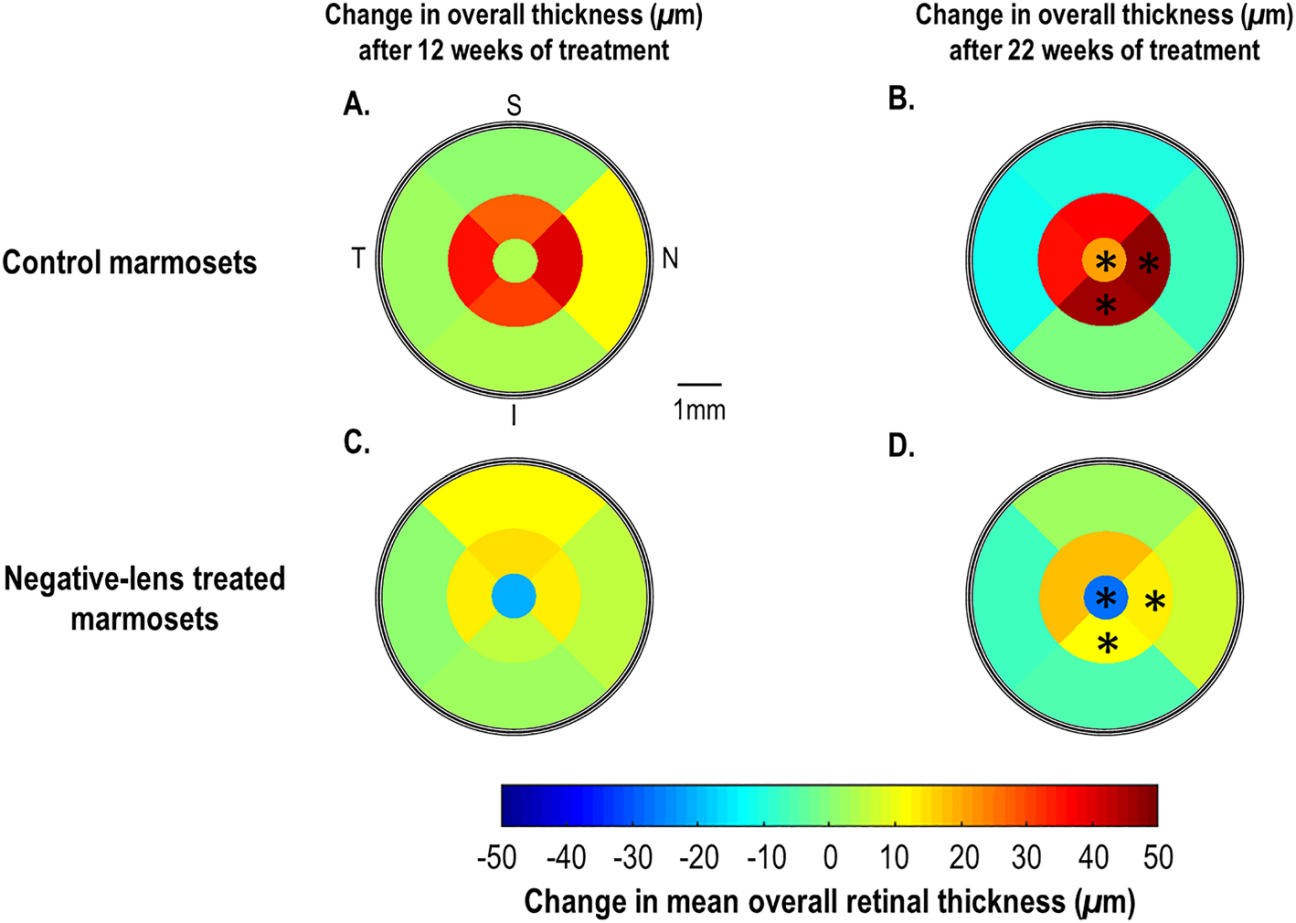
ETDRS color map summarizing the average 12-week change (**A** & **C**) and 22-week change in overall retinal thickness (**B** & **D**) for (**A** & **B**) control marmosets (*n* = 5) and (**C** & **D**) negative-lens treated marmosets (*n* = 8). Positive and negative values on color bar represent retinal thickening and thinning relative to baseline, respectively. S = Superior; T = Temporal; N = Nasal; and I = Inferior. Statistically significant difference in retinal thickness between control and negative-lens treated marmosets at the center, nasal inner and inferior inner ETDRS region is indicated by *p < 0.05 (in **B** & **D**).

Over the 22 weeks of follow-up, untreated control marmosets exhibited an overall age-related retinal thickening relative to baseline in all quadrants (overall change: + 17.27 ± 11.52 μm, *p* < 0.05; Fig. 6A,B) that was more prominent in the nasal and inferior regions (nasal region change: + 20.63 ± 15.08 μm, *p* < 0.05; inferior region change: + 22.38 ± 13.87 μm, *p* < 0.05).

In terms of eccentricity, as control marmoset eyes grew, their overall retina thickened significantly over time in the central (22-week thickness change: + 20.33 ± 13.97 μm; *p* < 0.05), and middle ring (+ 41.24 ± 17.83 μm; *p* < 0.05), and thinned in the outermost ring (− 7.47 ± 10.52 μm, *p* = 0.45).

The overall inner retina (RNFL-ONL) also thickened significantly with age (+ 22.73 ± 16.15 μm, *p* < 0.01), mainly due to significant thickening in the GCL (+ 2.07 ± 1.85 μm; *p* < 0.01), IPL (+ 5.57 ± 3.57 μm; *p* < 0.05), INL (+ 7.02 ± 2.70 μm; *p* < 0.05) and ONL (+ 10.78 ± 8.34 μm; *p* < 0.05). In the outer retina (− 3.80 ± 8.60 μm; *p* = 0.09), only the RPE changed and thinned significantly compared to baseline (− 1.70 ± 0.82 μm; *p* < 0.05).

### Effect of myopia development and progression in the marmoset retina compared to controls

**Figure 7:**
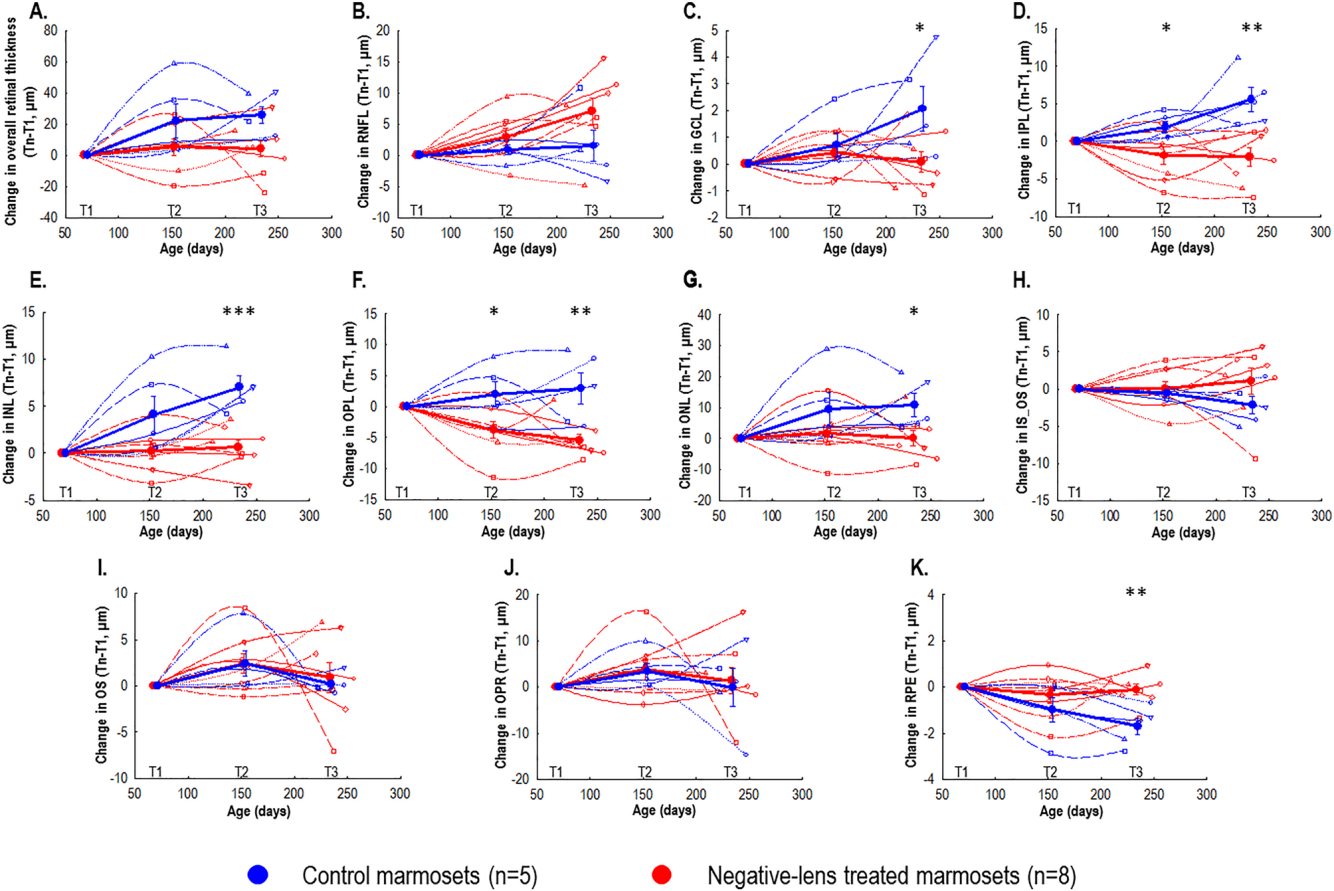
Change in mean overall retinal thickness (**A**) and the 10 individual retinal thickness layers (**B-K**) for control and negative-lens treated marmosets normalized to baseline over 22 weeks of treatment (T1: baseline, T2: 12 weeks of treatment, T3: 22 weeks of treatment). Thin symbols and lines represent data for each marmoset. Thick symbols and lines represent data for each treatment group. The untreated control group (*n* = 5) is shown with blue circles, and the negative-lens treated group (*n* = 8) is shown with red circles. Positive and negative values on the ordinate represent retinal thickening and thinning relative to baseline, respectively. Statistically significant difference at each treatment time point is indicated by *p < 0.05, ** *p* < 0.01 and ***p < 0.001. The data are shown as mean ± SE.

Treated marmosets’ overall retina thickness did not change significantly over the 22 weeks of treatment compared to the thickening experienced by controls (22-week change: + 3.46 ± 16.63 μm; controls: + 17.27 ± 11.52 μm, *p* = 0.13; Figs. 6C,D, Fig. 7A). However, assessing the individual thickness of each retinal layer (i.e. in all quadrants) identified significant differences between treated and control marmosets in six of the ten layers evaluated, most of which were located in the inner retina: GCL (22-week change, treated: + 0.08 ± 1.07 μm, control: + 2.07 ± 1.85 μm; *p* < 0.05; Fig. 7C), IPL (treated: − 2.08 ± 3.56 μm, control: + 5.57 ± 3.57 μm; *p* < 0.01; Fig. 7D), INL (treated: + 0.62 ± 2.17 μm, control: + 7.02 ± 2.70 μm; *p* < 0.01; Fig. 7E), OPL (treated: − 5.49 ± 3.02 μm, control: + 2.89 ± 5.62 μm; *p* < 0.01; Fig. 7F), ONL (treated: + 0.05 ± 6.97 μm, control: + 10.78 ± 8.34 μm; *p* < 0.05; Fig. 7G) and RPE (treated: − 0.12 ± 0.65 μm, control: − 1.70 ± 0.82 μm; *p* < 0.05; Fig. 7K). The mean retinal thickness changes (for all quadrants) in other retinal layers are summarized in Figs. 7B,H,I,J. Quadrant-specific differences in the thicknesses of these 6 retinal layers between control and treated marmosets are summarized in Table 1.

**Table 1:** Retinal quadrant-specific differences in the individual retinal layer thicknesses (mean ± SD) between the treated and control marmosets.

Individual retinal layer	Quadrant-specific differences between treated and control marmosets
GCL	Superior (treated: -0.95 ± 2.74 μm, control: 2.41 ± 1.98 μm)*
	Inferior (treated: -0.76 ± 1.90 μm, control: 3.84 ± 4.32 μm)*
IPL	Nasal (treated: -6.09 ± 6.44 μm, control: 4.57 ± 5.89 μm)*
	Temporal (treated: -0.11 ± 3.79 μm, control: 6.89 ± 3.43 μm)**
	Inferior (treated: -2.20 ± 2.91 μm, control: 2.61 ± 4.14 μm)*
INL	Temporal (treated: 2.18 ± 1.97 μm, control: 6.872 ± 2.65 μm)**
	Superior (treated: 2.38 ± 4.07 μm, control: 9.20 ± 4.49 μm)*
	Inferior (treated: -0.38 ± 3.21 μm, control: 4.55 ± 3.67 μm)*
OPL	Nasal (treated: -7.22 ± 4.19 μm, control: 2.29 ± 6.88 μm)**
	Temporal (treated: -2.96 ± 3.65 μm, control: 4.03 ± 4.78 μm)*
ONL	Nasal (treated: 6.01 ± 5.69 μm, control: 13.07 ± 5.47 μm)*
	Temporal (treated: -4.11 ± 6.74 μm, control: 6.13 ± 8.70 μm)*
RPE	Nasal (treated: -0.22 ± 0.64 μm, control: -1.58 ± 1.44 μm)*
	Temporal (treated: 0.04 ± 0.99 μm, control: -1.95 ± 1.22 μm)**
	Superior (treated: -0.06 ± 1.33 μm, control: -2.17 ± 0.34 m)**
	Inferior (treated: 0.40 ± 1.20 μm, control: -1.48 ± 1.01 μm)*

*p < 0.05; **p < 0.01.

**Figure 8:**
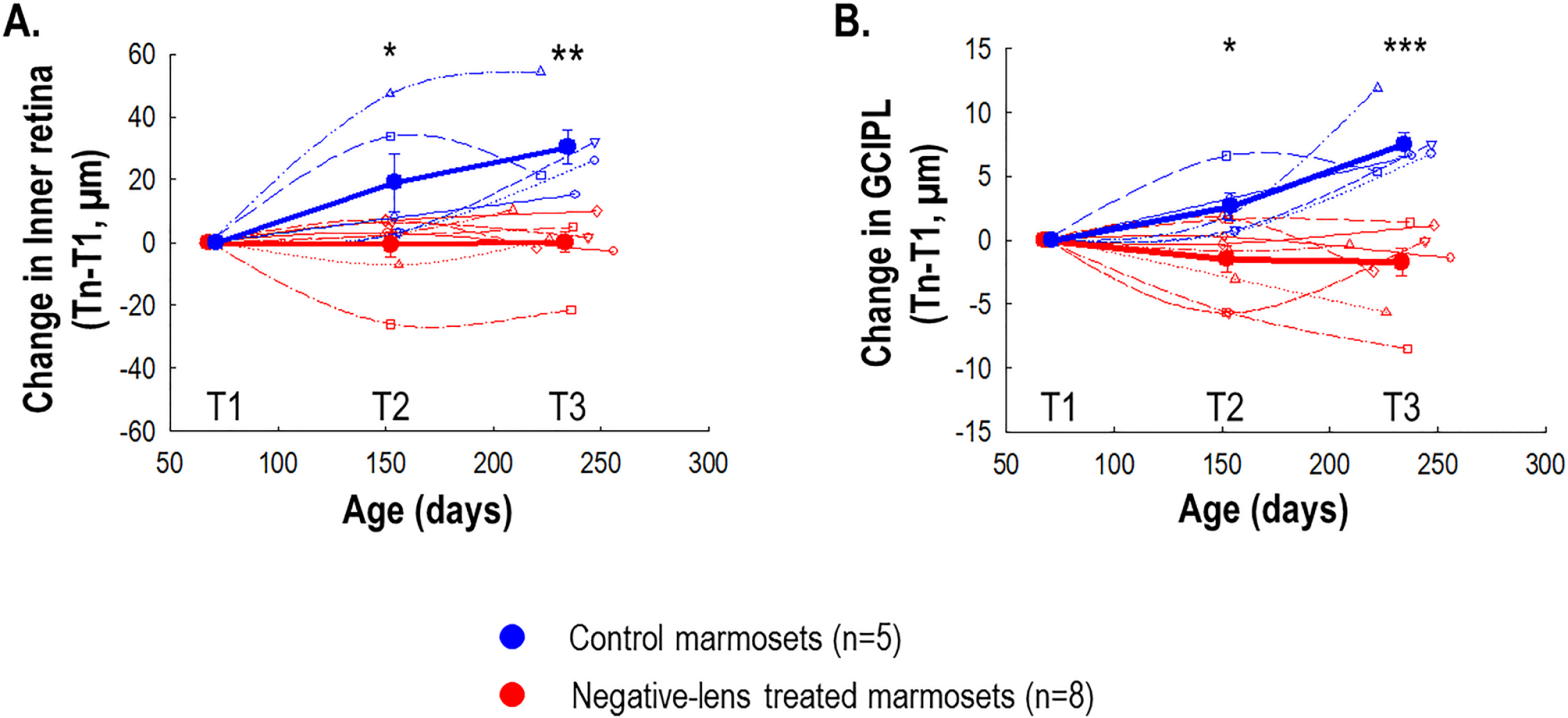
Thickness changes in the inner retina (**A**) and GCIPL (**B**) for control and negative-lens treated marmosets normalized to baseline over 22 weeks of treatment (T1: baseline, T2: 12 weeks of treatment, T3: 22 weeks of treatment). Thin symbols and lines represent data for each marmoset. Thick symbols and lines represent data for each treatment group. The untreated control group (*n* = 5) is shown with blue circles, and the negative-lens treated group (*n* = 8) is shown with red circles. Positive and negative values on the ordinate represent retinal thickening and thinning relative to baseline, respectively. Statistically significant difference at each treatment time point is indicated by *p < 0.05, ** *p* < 0.01 and ***p < 0.001. The data are shown as mean ± SE.

Treated eyes experienced significantly less thickening of the overall inner retina (22-week change, treated: + 0.26 ± 10.01 μm, control: + 29.83 ± 15.03μmp < 0.01; Fig. 8A) and thinning of the GCIPL (22-week change, treated: − 2.00 ± 3.47 μm, control: + 7.64 ± 2.51 μm; independent t-test, *p* < 0.001; Fig. 8B) that differed significantly from the thickening of controls. There was not a significant treatment effect on the outer retina (22-week change, treated: + 3.21 ± 15.80 μm, control: − 3.80 ± 8.60 μm; *p* = 0.39) or GCC (22-week change, treated: + 5.08 ± 8.45 μm, control: + 9.13 ± 5.31 μm; *p* = 0.36).

### Individual retinal layer thicknesses as predictors of refraction and eye growth

The changes in vitreous chamber depth and refractive error over the 22 weeks were well predicted by the thickness changes that took place in the most eccentric retinal locations of the GCL, INL, OPL, ONL and RPE assessed that corresponded to the near to mid peripheral retina (Stepwise regression model, Table 2).

**Table 2:** Stepwise regression model of the ocular parameters with thicknesses of ETDRS zone in individual retinal layers.

Dependent variables (change in VCD and Rx)	Independent variables (thickness change in each layer and ETDRS region)	R^2^	*p*-value
VCD	GCL nasal outer	0.64	0.001
	INL nasal outer + temporal outer	0.78	< 0.001
	OPL superior inner	0.49	0.008
	ONL nasal outer + inferior inner + superior inner	0.82	0.001
	RPE inferior outer	0.66	0.001
Rx	GCL nasal outer	0.48	0.009
	INL nasal outer + temporal outer + inferior outer	0.87	< 0.001
	OPL superior inner	0.50	0.007
	ONL nasal outer	0.52	0.005
	RPE inferior outer	0.80	< 0.001

When all significant independent variables from the stepwise regression models were combined in a multiple regression analysis, the regression value increased to *R*^2^ = 0.94 (*p* < 0.05) for growth (VCD change) rate and to *R*^2^ = 0.91 (*p* < 0.05) for refraction rate.

## Discussion

This study confirms that retinal thickness changes can be assessed and monitored longitudinally in marmosets non-invasively *in-vivo* in a safe, reproducible and reliable manner using SD-OCT^37–39^. The thickness template of the central-mid peripheral retina was monitored for 5.5 months in a non-human primate model of emmetropization in normal growing eyes compared to eyes induced with myopia. The retina of untreated eyes thickened as eyes grew and emmetropized. Myopic eyes, on the contrary, experienced significantly less retinal thickening and thinning at times as eyes grew faster, which occurred mostly in the near to mid-peripheral regions of the inner retina and GCIPL.

The non-invasive nature of the OCT permits characterizing ocular conditions over time and optimizes the use of animals and tissue compared to *in-vitro* techniques. This study describes the longitudinal changes that occurred in the central and near-mid peripheral retinal thickness profiles of emmetropic and myopic marmosets using SD-OCT. The OCT scan acquisition and segmentation methods used for this work provided marmoset retinal scans that had a small variability and high reproducibility, equivalent to those in rhesus macaques^40^ and tree shrews^19,41^. This study is significant due to the similarities between marmoset and human eyes, which makes these findings highly translatable^42^.

Before treatment started, marmoset retinas were thinnest at the fovea, and thickened with eccentricity. This thickness topography is comparable to humans and derives from mechanical forces remodeling the central retina during development. During mid-gestation, centripetal displacement of all retinal cells results in a very thick central retina before the fovea is formed. After mid-gestation and continuing through early postnatal retinal development, centrifugal displacement of ganglion cells and neurons of the INL, and further inward displacement of photoreceptors, create a shallow foveal pit and thicker para and perifoveal thickness^43^. As expected, the nasal quadrant carrying the papillomacular bundle was the thickest, followed by the inferior and superior quadrants, where the arcuate retinal nerve fibers can be found. The temporal quadrant was the thinnest. The retinal thickness profiles observed in this study in marmosets are similar to studies providing normative OCT data for healthy pediatric populations^44,45^ and adult cynomolgus monkeys^46^. The most significant difference is that in children and adult monkeys, the retinal thickness in the middle ETDRS ring appears thicker than the outermost ETDRS ring. This is likely due to differences in the areas assessed by the ETDRS rings: the outermost ring in this study covers the near to mid-peripheral areas in the marmoset retina while in humans, this same outermost ring dimension will only cover the perifovea. These findings provide an important normative retinal thickness database for studies exploring the effects of disease processes on the marmoset retina.

Untreated control marmosets emmetropized while marmosets treated with negative lenses developed axial myopia. After birth, marmosets are known to emmetropize to mild hyperopia or a small amount of myopia^47^ which occurs in parallel with a reduction in refraction variability, a hallmark of emmetropization^48^. Emmetropization is an active dynamic process of postnatal ocular development aimed to match the ocular power of the eye to its axial length. Treated marmosets were exposed to hyperopic defocus from the negative lenses and responded by increasing their eye growth in an effort to match axial length to the imposed defocus. The mechanism of emmetropization has been confirmed in a wide range of animal species including fish^49^, chicks^50,^ mice^51^, guinea pigs^52^, tree shrews^53^, common marmosets^54^ and rhesus monkeys^55^, corroborating that emmetropization is an evolutionary conserved process, and highlighting the role of the visual experience in the homeostatic control of eye growth.

In this study, untreated eyes experienced retinal thickening as they grew older over a period of 5.5 months, primarily due to a significant thickening of the GCL, IPL, INL and ONL, all located in the inner retina. This pattern indicates a relative thickening of the retinal plexiform and nuclear layers with normal eye growth and emmetropization. While most of the mammalian retinal morphology and function develops before eyes open^56^, there is further growth-related retinal changes that occur after eye opening^57^. These postnatal changes are known to be guided by visual and cellular activity^58^. During postnatal mammalian retinal development, a thinning of the retinal nuclear layers and a thickening of the synaptic layers has been described, which is attributable to a redistribution of existing cells and reorganization of synaptic connections of the retina as it grows and expands^57^. Although we did not observe thinning of nuclear layers during the follow-up period of this study, it is expected that further maturation of the retina as marmosets develop further may yield these changes.

Myopic eyes also experienced a retinal thickening after 5.5 weeks of treatment, but the thickening degree was less than in control eyes. These differences were attributable to thickness changes in the GCL, IPL, INL, OPL and ONL, all located in the inner neuroretina. During the development of axial myopia, eyes grow at faster rates, and they experience greater tissue stretch than normal growing eyes^59^. Thinner inner retinas have been described in myopic human^15^, chick^17,18^ and tree shrew eyes^19^. Most of the retinal thinning observed in the myopic marmosets was localized in the post-photoreceptor inner retina, supporting the hypotheses by Atchison et al.^60^ and Chui et al.^61^ proposing a passive retinal stretching accompanied by post-photoreceptor cell changes as the biological basis for reduced visual function observed in human myopes. Of particular importance is the significant thickness changes we observed in the ONL, OPL, INL and IPL as these may explain the observed reduction in the b- and a-wave described in myopes in the past^62–64^. Image quality changes caused by the imposed defocus might also affect the visual input and activity required for adequate synaptic connections. Visual stimulation is required for the regulation of ganglion cell synapses and dendritic ramification. In its absence, there is an impairment of this developmental process^65^. While there is evidence of significant IPL thinning in high myopic form-deprived tree shrews using OCT and histology measures^19^, there is also evidence of an expansion of the ganglion cell dendritic arbors in myopic form-deprived chicks. Such expansion of the ganglion cell dendritic arbor was observed in retinal flatmounts along the horizontal surface plane and appears aimed to maintain the bipolar cell to ganglion cell convergence ratio^66^.

While both groups had similar RPE thickness at baseline, myopic marmosets exhibited a relatively lower degree of RPE thinning than controls. Due to the very limited proliferation of primate RPE cells after birth^67^, the changes observed might be due to differences in RPE cell distribution, which have been described in multinucleated cells of myopic quokka wallabies^68^. In addition, the RPE mitochondria of myopic chick eyes appear to grow in size soon after occlusion, and return to normal after recovery^69^. Similar thickness changes have been described in the chick model of ocular growth^70^. How these RPE changes affect the overall retinal health of the myopic retina prior to the development of myopic pathology needs to be investigated.

Myopic marmosets also exhibited a thinning of the GCIPL and GCL compared to controls. Macular GCIPL and GCC thickness are parameters that include approximately 50% of retinal ganglion cells^71^, making them good clinical diagnostic markers for glaucoma comparable to circumpapillary RNFL^72,73^. In fact, central retinal ganglion cell loss in experimental glaucoma has been shown to be identical to that observed in the peripheral retina of monkeys^74^. Since similar macular GCIPL has been documented in high myopic human eyes^75,76^, these changes might be a product of the mechanisms that predispose myopic eyes to glaucomatous remodeling. However, they might also be myopic the result of myopic remodeling and confounding markers wrongly interpreted as glaucomatous damage in healthy myopic eyes. With the marmoset model being increasingly used in neuroscience, ocular disease and therapy research^42,77^, studies exploring glaucoma in marmosets^78,79^ may find macular GCIPL and GCL thinning to be an important marker to monitor glaucomatous remodeling in myopic eyes.

The statistical models performed confirmed the longitudinal association between the enlargement of the eye and the thinning of the near-mid peripheral retina. Analogous trends of increasing foveal and parafoveal retina thicknesses with age have been described in young and adult marmosets^39^. The foveal region is a developmentally advanced area that supports a complex connection and organization template of retinal cells and processes^80^, making the central retina more resistant to growth and stretch than the peripheral retina^81^. To maintain high visual acuity in the central retina during normal postnatal ocular growth, the central retina is less responsive to expansion in order to maintain high photoreceptor and ganglion cell density. The axial elongation taking place during myopia development and progression possibly affects neural and sampling functions and may explain the reduced visual performance in the mid to far periphery of myopic eyes^60,61^, which agrees with studies suggesting peripheral stretching in human myopic eyes^20,82^.

The results from this study strongly suggest that the individual retinal layers of myopic eyes undergo thickness changes that differ from normal growing eyes and are associated with increased axial elongation. While the distinctive effect of myopic growth on the individual layers of the retina might be due to differences in the biomechanical properties of retinal cells^83^, the selective thinning might not be solely attributed to passive retinal stretching^18^. In fact, refractive and axial length changes have not fully explained the reduced retinal function observed in human myopes^84^. Thus, the retinal thickness changes observed in the myopic retina may be due to the modified visual experience caused by defocus and subsequent altered neural responsiveness and not necessarily the accompanying axial elongation. This possibility needs further investigation as pointed out by Troilo et al.^66^.

While the magnitude of the changes might fall within the repeatability and axial resolution range of the instrument used, the sample size calculations performed during the design phase of the study confirmed that 12 marmosets were enough to provide a power of 80% and *α* = 0.05, which confirms that the distribution of data in control and treated animals is different and not occurring by chance. The differences identified in this non-human primate model of myopia in the GCL, IPL, INL, OPL, ONL and RPE between myopic and control marmosets are important to help understand the mechanisms of eye growth. However, the scale of the changes need to be validated longitudinally in human eyes prior to confirming its clinical relevance.

In summary, in vivo SD-OCT measurements can be performed safely and reliably to detect growth and myopia related changes in marmoset retinas. The longitudinal characterization of the thickness changes occurring in the retina of myopic eyes using SD-OCT confirms the existence of an overall retinal thinning occurring mostly in more eccentric locations of the inner retina possibly attributable to the mechanical stress exerted on the underlying retinal tissue that occurs as myopic eyes grow larger.

## Data Availability

The datasets generated during and/or analyzed during the current study are available from the corresponding author on reasonable request.
